# Geographical variation in the chemical profile and antimicrobial activity of *Solidago gigantea* essential oils

**DOI:** 10.3389/fmicb.2026.1740133

**Published:** 2026-02-09

**Authors:** Rita Filep, David U. Nagy, Edit Ormai, Béla Kocsis, Margita Szilágyi-Utczás, Dragica Purger, Viktória Lilla Balázs

**Affiliations:** 1 Department of Pharmacognosy, Faculty of Pharmacy, University of Pécs, Pécs, Hungary; 2 Department of Plant Evolutionary Ecology, Faculty of Biological Sciences, Institute for Ecology, Evolution and Diversity, Goethe University Frankfurt, Frankfurt am Main, Germany; 3 Department of Medical Microbiology, Medical School, University of Pécs, Pécs, Hungary; 4 Center for Sports Nutrition Science, Hungarian University of Sports Science, Budapest, Hungary

**Keywords:** biofilm, essential oil, giant goldenrod, terpenoids, water-steam distillation

## Abstract

**Introduction:**

The antibacterial significance of the essential oil of *Solidago gigantea*, a North American native species invasive in Europe, remains poorly understood. In this study, we investigated the chemical composition of *Solidago gigantea* essential oils (SEO) collected from three distinct Hungarian locations and evaluated their antibacterial activities against *Escherichia coli* ATCC 25922, methicillin-resistant *Staphylococcus aureus* (MRSA) ATCC 25923, and *Pseudomonas aeruginosa* ATCC 27853.

**Methods:**

Essential oils (SEO1, SEO2, SEO3) were obtained from inflorescences of *Solidago gigantea* by water-steam distillation. Chemical profiles were determined using gas chromatography–mass spectrometry (GC–MS). Antibacterial activity was assessed by broth microdilution, biofilm inhibition, and membrane damage assays, while scanning electron microscopy was used to visualize bacterial cell alterations.

**Results:**

A total of 110 constituents were identified, and relative quantities of certain components varied among samples. The major components included cyclocolorenone (10.46–29.69%), *α*-pinene (5.09–12.41%), α-gurjunene (2.76–6.32%), and bornyl acetate (4.31–6.06%). Minimum inhibitory concentration tests revealed that *E. coli* was the most susceptible (0.31–0.62 mg/mL), MRSA showed intermediate sensitivity (0.62–1.25 mg/mL), and *P. aeruginosa* was the most resistant (1.25–2.50 mg/mL). Biofilm assays showed strong inhibitory effects: *E. coli* biofilms were reduced by up to 95.7%, MRSA by over 90%, and *P. aeruginosa* by 87.3%. Synergistic interactions were observed between SEO and gentamicin against *P. aeruginosa*, and between ceftriaxone and the oil against *E. coli*. The essential oil’s effectiveness varied by sampling location, with the SEO3 sample showing the strongest antimicrobial activity, including the highest biofilm inhibition and over 80% membrane disruption in all bacterial species after 60 min.

**Conclusion:**

The essential oil of *Solidago gigantea* showed antibacterial and biofilm-inhibitory activity, influenced by the plant’s geographical origin.

## 1 Introduction

The overuse and misuse of antibiotics, together with natural evolutionary processes, have driven the global rise and spread of antibacterial resistance (Akova, 2016; Bhojyawal et al., 2024; Tahmasebi et al., 2025). A major contributor to this resistance is biofilm formation (Luo et al., 2021; Li et al., 2023), in which surface-attached microbial communities are encased in a self-produced extracellular matrix and exhibit survival rates hundreds to thousands of times higher than planktonic bacteria when exposed to antibiotics (Ceri et al., 1999; Costerton et al., 1999; Zhang et al., 2022; Michael and Waturangi, 2023). The escalating resistance problem highlights the need for integrative strategies that combine rational antibiotic use with novel adjunctive therapies (Wei et al., 2025). In this context, essential oils (EOs) have gained attention as promising candidates due to their antimicrobial properties and potential effectiveness against resistant pathogens (Veras et al., 2012; Raut and Karuppayil, 2014; Balázs et al., 2022, 2025; Piasecki et al., 2023).

Essential oils are volatile, natural, and complex mixtures of low-molecular-weight compounds, biosynthesized as secondary metabolites by aromatic plants. These metabolites are typically produced in response to biotic stressors such as herbivory and microbial attack (Raut and Karuppayil, 2014). Essential oils, either as monotherapies or in synergistic combinations, have demonstrated substantial pharmacological properties, indicating their potential utility in the treatment of both infectious and non-infectious diseases (Raut and Karuppayil, 2014; Khwaza and Aderibigbe, 2025). On the other hand, several factors affect the yield and quality of EOs in plants, including environmental conditions, with climatic and edaphic factors being particularly influential and often linked to geographic location (Figueiredo et al., 2008; Msaada et al., 2009; Rahimmalek et al., 2009; Sharma et al., 2016; Herlina et al., 2017; Mehalaine and Chenchouni, 2021). Climatic variables such as temperature, precipitation, humidity, and solar radiation can significantly alter both the quantity and chemical composition of EOs (Hussain et al., 2010; Silva et al., 2016; Mollaei et al., 2020; Santos et al., 2025). Seasonal changes and differences in microclimate have been reported to modulate the biosynthesis of chemical compounds, leading to variations in chemical profiles among populations of the same species grown in different regions (Rahimmalek et al., 2009; Filep et al., 2016; Balázs et al., 2025; Santos et al., 2025). For instance, Rahimmalek et al. (2009), in their investigation of the EO composition of 19 accessions from six different *Achillea* species, reported a high degree of inter- and intra-specific chemical polymorphism, attributing this variation to genetic and environmental factors as well as their interaction.

*Solidago* species have long been utilized in European traditional medicine and are recognized as rich sources of phenolic compounds with diverse pharmacological activities, particularly in the complementary management of various inflammatory disorders of the urinary tract (Fursenco et al., 2020; Marksa et al., 2020). In recent years, scientific interest in the bioactive constituents of *Solidago* species has increased, with a special focus on their EOs due to their complex chemical profiles and promising biological properties (Thiem and Goślińska, 2002; Huang et al., 2012; Li et al., 2012; Deng et al., 2015; Abdel Motaal et al., 2016; Liu et al., 2016; Jasicka-Misiak et al., 2018; Barczewski et al., 2024; Bisht et al., 2024). Among these, *Solidago gigantea* (Aiton), a North American native species with a circumpolar distribution extending from Europe to Asia (Weber and Jakobs, 2005), has attracted particular attention. Although it predominantly inhabits mesic and wet environments in its native range, in Europe it has become highly invasive, successfully colonizing wetlands, disturbed mesic and drier sites, as well as tree plantations, where it often forms dense, mono-dominant stands (Török et al., 2003; Weber and Jakobs, 2005; Pal et al., 2015; Nagy et al., 2022).

The chemical composition of *Solidago gigantea* essential oil (SEO) has been reported in several studies, with *α*-pinene, bornyl acetate, spathulenol, isospathulenol, (−)-cyclocolorenone, and p-cymene identified as the main constituents (Kalemba et al., 2001; Kalemba and Thiem, 2004; Radušienė et al., 2022). For instance, Benelli et al. (2019) investigated EOs obtained from different plant parts (leaves, inflorescences, and roots) of Hungarian *Solidago gigantea* as a potential source of botanical insecticides, identifying a total of 80 constituents. While research on the antibacterial activity of *Solidago* species EOs is increasing (Mishra et al., 2020; Abdulkareem et al., 2023; Bisht et al., 2024; Malićanin et al., 2024), data specifically on *Solidago gigantea* remain limited. Among the few available studies, Kołodziej et al. (2011) reported that ethanolic extracts of *Solidago gigantea* exhibited relatively strong activity against Gram-positive bacteria, while Gram-negative strains were less affected. Moreover, most existing studies are based on samples collected from a single habitat and do not consider potential geographical variation in EO composition.

In this study, we sought to obtain a better understanding of antibacterial effects of EO derived from the inflorescence of *Solidago gigantea*, collected from three wild populations in Hungary. The aims of this study were as follows: (1) To determine EO yield and chemical composition *Solidago gigantea* inflorescences using water-steam distillation and GC–MS. (2) To evaluate the antibacterial and biofilm-inhibitory activities of the SEOs against opportunistic, biofilm-forming pathogens such as *Escherichia coli*, *Pseudomonas aeruginosa*, and methicillin-resistant *Staphylococcus aureus* (MRSA). (3) To evaluate the combined effects of the SEO and selected antibiotics through synergistic interaction studies. (4) To assess whether SEO obtained from different geographic location differ in yield, chemical composition, antibacterial and biofilm-inhibitory activities.

## 2 Materials and methods

### 2.1 Plant material and environmental parameters of collection sites

**Figure 1:**
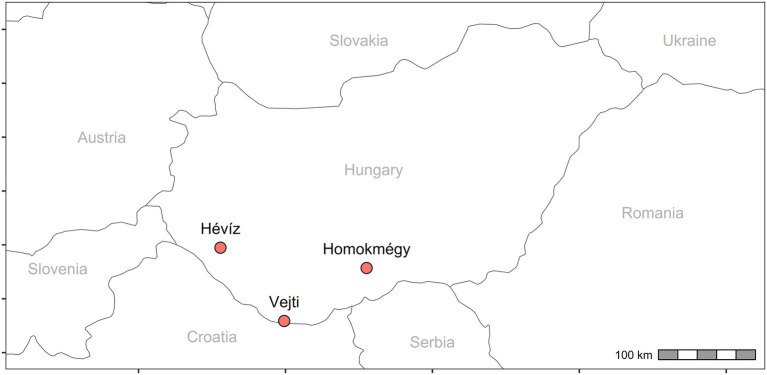
*Solidago gigantea* sampling sites.

We collected *Solidago gigantea* inflorescences by using a pruning shears in the peak flowering phenophase during a 2 weeks period in August 2022, from three locations in Hungary: Hévíz (SEO1): 46.4737 N, 17.1150 E, elevation: 109 m above sea level; Homokmégy (SEO2): 46.2854 N, 19.1044 E, elevation: 86 m above sea level; and Vejti (SEO3): 45.793542 N, 17.982658 E, elevation: 91 m above sea level (Figure 1). To minimize variation related to ontogeny and plant condition, inflorescences were collected from multiple healthy, mature individuals per population within the same phenological stage. Weather conditions were sunny and slightly windy, and there was no rainfall for 48 h before each sampling day. The samples were air-dried separately, at 22–24 °C in a storage room, without direct light, for one month.

Nineteen bioclimatic variables for the *Solidago gigantea* collection sites were obtained from the WorldClim version 2.1 database (Fick and Hijmans, 2017) for the available 1971 to 2000 period, at spatial resolutions of 30 s (~1 km^2^). These variables were included to provide a descriptive climatic context for the sampled populations; given the limited number of populations (n = 3), no statistical analyses of climatic differences were performed.

### 2.2 Essential oil extraction – distillation conditions

Essential oil was isolated from the inflorescence part of the *Solidago gigantea* via water-steam distillation. Dry plant material was cut into small pieces and inserted in 10-L flasks filled with 5–6 L of distilled water, then subjected to water-steam distillation using a Clevenger-type apparatus for 3 h (at 175 °C). The SEO was decanted, separated from water and dehydrated using anhydrous Na_2_SO_4_. They were stored in amber vials capped with PTFE-faced silicon septa at 4 °C until analyzed. An EO distillation apparatus official in Pharmacopoea Hungarica VIII was used during our investigation. During the process, a short-necked ground round-bottomed flask was used, the inner diameter of which at the flared end of the ground is 29 mm. The inner diameter of the flared end of the distillation attachment is 10 mm. The distillation attachment is a custom-made design. The heating basket is FALC type, MA series (Scharlab Hungary Ltd.).

### 2.3 Determination of essential oils composition

Semi-quantitative chemical analysis of the EOs from the inflorescence of the *Solidago gigantea* was performed by using gas chromatography–mass spectrometry (GC–MS). The analytical parameters for GC–MS analysis were as follows: the solvent (DCM), the n-alkane homologous series (C7-C30; Supelco #49451-U), and the samples at a 1:1000 dilution were analyzed on an Agilent 7890B gas chromatograph coupled to an Agilent 7010B triple quadrupole mass spectrometer in MS1 scan and EI (electron ionization) mode. A HP-5 ms (5% diphenyl + 95% dimethyl polysiloxane) 30 m × 0.25 mm ID × 0.25 μm column was used for separation. The gas chromatograph was equipped with a split/splitless injector. The carrier gas was He (5.0 purity). The additional setup parameters are listed in Table 1.

**Table 1:** The gas chromatography–mass spectrometry setup parameters.

Parameter	Setting
Reference series used for LRI calculation	*n*-alkane
Temperature program	
Initial temperature	50 °C
Temperature gradient	3°C/min
Final temperature	350 °C (5 min)
Injection temperature	280 °C
Constant linear velocity	30.0 cm/s
Interface temperature	200 °C
Ione source temperature	250 °C
Acquisition mass range	40–550 m/z

Compounds were identified using the FFNSC 4.0 GC–MS library (Shimadzu, Kyoto, Japan), which is based on a standardized and pre-validated analytical protocol. The library provides rigorous specifications for GC and MS operating parameters, column type, temperature program, and retention index calculation using a homologous n-alkane series. Method repeatability and spectral reproducibility were validated during the development of the FFNSC 4.0 library. In this study, all analyses were conducted in strict accordance with the library instructions to ensure reliable compound identification. The MS spectrum similarity criterion was >90%, while the linear retention index (LRI) window was ± 5 units. Semi-quantitative results were expressed as relative area percentages based on GC–MS total ion current (TIC) peak areas without any further correction. Compound nomenclature was based on the FFNSC 4.0 mass spectral library.

### 2.4 Microbiological assay

#### 2.4.1 Bacterial strains and cultivation conditions

Three human pathogenic bacterial strains were used in this study. The Gram-negative strains *Escherichia coli* ATCC 25922 and *Pseudomonas aeruginosa* ATCC 27853 are common mucosal colonizers and frequent causes of nosocomial infections, particularly in immunocompromised individuals (Engelhart et al., 2002; Garcia-Vidal et al., 2018; Krell and Matilla, 2024; Liu et al., 2024). While *E. coli* is part of the normal intestinal flora, it may become pathogenic under immunosuppressed conditions, often as extended-spectrum *β*-lactamase (ESBL)-producing strains (Liu et al., 2024). The Gram-positive strain methicillin-resistant *Staphylococcus aureus* (MRSA) ATCC 25923 commonly colonizes the skin and mucosal surfaces and represents a major risk factor for invasive infections in immunosuppressed populations (Silva-Santana et al., 2021). The pathogens were cultured using Brain Heart Infusion (BHI) Broth (Sigma-Aldrich Ltd., Budapest, Hungary) as the liquid medium. The bacterial suspensions were incubated in a shaking incubator (C25 Incubator Shaker, New Brunswick Scientific, Edison, NJ, USA) at 37 °C and 60 rpm for 12 h. Before microbiological tests, the exact germ count was determined using spectrophotometric measurement.

#### 2.4.2 Stock solutions containing *Solidago gigantea* oil

Due to the hydrophobic nature of EOs, a stock solution incorporating an emulsifier was prepared prior to all microbiological assays. Tween40 was selected as the emulsifying agent. *Solidago gigantea* EO was dissolved in Brain Heart Infusion (BHI) medium supplemented with 1% Tween40, in accordance with the specified *in vitro* protocol. To ensure that the emulsifier itself exerted no antibacterial, anti-biofilm, or membrane-degradation effects, a control containing only the emulsifier was included in all experiments. The results demonstrated that 1% Tween40 had no significant impact on the outcomes of the assays.

#### 2.4.3 Determination of minimal inhibition concentration

The minimum inhibitory concentration (MIC) of the SEOs were determined using the microdilution method in accordance with CLSI guidelines [CLSI Document M 100-S22; (Sewell et al., 2025)]. The MIC was defined as the lowest concentration of the EO that completely inhibited visible bacterial growth in all parallel replicates. The procedure was carried out on 96-well microtiter plates, and, to ensure accuracy, all tests were performed in 8 replicates. Bacterial suspensions were adjusted to a concentration of 10^5^ CFU/mL. Subsequently, 100 μL of the bacterial suspension and 100 μL of the SEOs at various concentrations were added to each well. Stock solutions from the SEOs (5 mg/mL) were prepared in Brain Heart Infusion (BHI) using 1% Tween40 as emulgent and serial two-fold dilution was made up to (0.0390 mg/mL). The plates were incubated at 37 °C for 24 h, and then absorbance values were measured at 600 nm using a microplate reader (BMG Labtech, Budapest, Hungary). Strain-specific antibiotics were included as positive controls. In the case of *E. coli*, ceftriaxone (40 mg/mL stock, Hospira, 250 mg powder), in the case of MRSA and *P. aeruginosa*, gentamicin (Gentamicin Sandoz 80 mg/ 2 mL injection, Sandoz) antibiotics were used.

#### 2.4.4 Biofilm inhibition assay

Crystal violet (CV) assay was employed to evaluate the biofilm inhibitory activity of water-steam distilled EOs from the inflorescence of *Solidago gigantea* against the three bacterial strains investigated in this study. Biofilm formation was modeled using a 96-well microtiter plate assay, an approach commonly employed to investigate early-stage biofilm development. A volume of 200 μL of bacterial suspension, containing approximately 10^8^ CFU/mL, was added to each well. The plate was incubated at 37 °C for 24 h to allow for biofilm development, and after that non-adherent cells were removed by washing with 0.9% physiological saline. Subsequently, MIC/2 concentrations determined in previous MIC studies of the SEOs (Supplementary Table S1) were added to the pre-formed biofilms. The plates were then incubated for an additional 24 h at 37 °C. After this period, the wells were washed with distilled water to remove residual compounds. The adhered cells were fixed with 99% methanol (Molar Chemicals Ltd., Halásztelek, Hungary) for 15 min at room temperature (23 °C). After removing the methanol, the biofilms were stained with 0.1% CV for 20 min. The CV binds to negatively charged molecules in the extracel lular matrix of biofilms, enabling the quantitative determination of total biofilm biomass. The CV dye bound to the biofilm was dissolved with 200 μL of 33% acetic acid solution, thus ensuring a suitable homogeneous solution for absorbance measurement. Absorbance was measured at 595 nm using a SPECTROstar Nano microplate reader (BMG Labtech, Budapest, Hungary) (Peeters et al., 2008; Kerekes et al., 2013). Controls included a positive control (bacterial suspension only), a negative control (nutrient broth only), and a solvent control (bacterial suspension in nutrient broth containing 1% Tween 40) to account for any potential inhibitory effects of the emulsifier (Peeters et al., 2008).

#### 2.4.5 Membrane degradation assay

Membrane damage was assessed by quantifying the release of intracellular DNA into the extracellular environment. Both concentration- and time-dependent assays were conducted to evaluate the membrane-disruptive effect of the SEOs. For each assay, bacterial suspensions with a density of 10^8^ CFU/mL were prepared in phosphate-buffered saline (PBS). In the concentration-dependent experiment, bacterial cells were exposed to SEOs at different concentrations (MIC/4, MIC/2, MIC, MIC×2, MIC×4; Supplementary Table S1) for one hour. In the time-dependent experiment, cells were treated with SEOs at 2 × MIC and incubated for varying durations: 0, 10, 20, 40, 60, and 90 min. In both assays, untreated bacterial suspensions served as positive controls. Following the treatments, samples were centrifuged at 12,000 × g for 2 min (LabTech 624R, Labtech Ltd., Hungary). The absorbance of the nucleic acid-containing supernatant was measured at 260 nm using a spectrophotometer (Jasco V-730BIO, Able-Jasco Ltd., Hungary). The amount of DNA released from the cells was used to infer membrane damage. The higher the amount of nucleic acid leaked, the greater the extent of membrane damage. The results are expressed as a percentage compared to the control. The MIC×4 concentration was used as a positive reference to induce pronounced membrane disruption and to differentiate membrane damage-associated DNA release from growth inhibition effects.

#### 2.4.6 Synergistic effect study – checkerboard titration

Synergistic interactions between antibiotics and SEOs were assessed using the checkerboard titration method, which serves as an initial screening approach to identify potential synergistic effects. Assays were conducted in 96-well microtiter plates with eight replicates per condition, using bacterial cultures standardized to 10^5^ CFU/mL. According to the previously determined MIC, emulsions were prepared using BHI medium and 1% Tween40 (Sigma-Aldricht Ltd.). In each study arrangement, a halving dilution series was prepared at concentrations corresponding to predetermined MIC values, which concentrations were as follows: MIC×2, MIC, MIC/2, MIC/4 MIC/8. Subsequently, according to the test layouts, 100 μL of bacterial suspension and then 50 μL of SEO (substance A) and 50 μL of antibiotic (substance B) were applied to the cells of the microtiter plates: substance A: SEO3, substance B: for *P. aeruginosa* – gentamicin, for MRSA – vancomycin, for *E. coli* – ceftriaxone. In parallel, a bacterial suspension without SEO was applied to a microtiter plate as a positive control, and only BHI medium as a negative control. The prepared microtiter plates were incubated (24 h, 37 °C), and then their absorbance was measured at a wavelength of 600 nm with a plate reader (BMG Labtech, Bio-Tek Ltd.). Following the test, combination MIC values were calculated. For this, the average of the absorbances of the parallel tests in each layout was calculated. The concentration at which the absorbance of the positive control was reduced to ±10% was considered the combination MIC value. From the combination MIC values calculated in this way, the fractional inhibitory concentration index (FICI) was calculated, which is a dimensionless numerical value that helps to evaluate the combination effect of pharmacons. The interaction between the EO and antibiotics was interpreted based on the FICI as follows: FICI ≤ 0.5 was considered synergistic, FICI >0.5–1 additive, FICI >1–4 indifferent, and FICI >4 antagonistic (Odds, 2003).


$$\begin{array}{l} \text{FICI}=(\mathrm{MIC}\;A\;\text{combination})/(\mathrm{MIC}\;A)\\ +(\mathrm{MIC}\;B\;\text{combination})/(\mathrm{MIC}\;B) \end{array}$$

### 2.5 Statistical analysis

All statistical analyses were performed using R version 4.4.1 (R Core Team, 2024). The effects of SEOs on the bacterial biofilm inhibition rate were analyzed as inhibition rate (%), and was evaluated using linear and generalized linear mixed-effects models with the lme4 package [version 1.1–29; Bates et al., 2015]. In the first analysis, we evaluated the interactive effects of bacterial species (*E. coli*, *P. aeruginosa*, and MRSA) and SEO origin (three samples sites) on the inhibition rate. The model included bacterial species, SEO samples, and their interaction as fixed effects. In the second analysis, we tested the inhibition rate of biofilm formation separately for each bacterial species, using the SEO samples and SEO concentration as fixed effects and their interaction. In the third analysis, the interactive effects of SEO population and treatment duration were assessed on the inhibition rate, again separately for each bacterial species.

Random effect were included to account for replicate effects where applicable. Response variables were log-transformed when necessary to meet model assumptions of normality and homoscedasticity. Type III ANOVA was performed using the Anova() function from the car package (Fox and Weisberg, 2019), to evaluate the significance of main effects and interactions. For pairwise comparisons, estimated marginal means were calculated using the emmeans R package (Lenth, 2023), and *p*-values were adjusted with the Tukey method for multiple comparisons. A p-value < 0.05 was considered statistically significant.

## 3 Results

### 3.1 Yield of *Solidago gigantea* essential oil

The EO yields of *Solidago gigantea* inflorescences obtained from different locations in Hungary showed differences. The highest EO yield was recorded in the sample from Hévíz (1.89 μL·g^−1^), whereas the lowest yield was observed in the sample from Homokmégy (1.69 μL·g^−1^) (Table 2).

**Table 2:** Essential oil yields from the inflorescence of *Solidago gigantea* collected from three distinct geographical locations in Hungary.

Samples	Total measured quantity (g)	Obtained SEO volume (μL)	SEO yield (μL·g^−1^)
SEO1	190.30	360	1.89
SEO2	118.40	200	1.69
SEO3	147.04	270	1.83

SEO1, Hévíz; SEO2, Homokmégy; SEO3, Vejti.

### 3.2 Climatic parameters

Differences were observed among the three *Solidago gigantea* collection sites in terms of bioclimatic variables. Regarding temperature-related parameters (e.g., annual mean temperature, mean diurnal range, maximum temperature of the warmest month), the highest values were recorded at the southernmost site, SEO3, while the other two sites exhibited similar temperature profiles. Precipitation was lowest at SEO2 compared to the other sites, whereas the highest mean annual precipitation was observed at SEO1 (Table 3).

**Table 3:** Nineteen bioclimatic variables for the *Solidago gigantea* collection sites (WorldClim database).

Climate	SEO1	SEO2	SEO3
Annual mean temperature	10.4129	10.9739	11.2089
Mean diurnal range (Mean of monthly (max temp – min temp))	9.5966	9.5983	9.7560
Isothermality (Mean diurnal range/Temperature Annual Range) (×100)	0.3166	0.3132	0.3205
Temperature seasonality (standard deviation ×100)	0.0256	0.0265	0.0257
Max temperature of warmest month	26.3097	27.1742	27.2226
Min temperature of coldest month	−3.9967	−3.4645	−3.2161
Temperature annual range (max temperature of warmest month – min temperature of coldest month)	30.3064	30.6387	30.4387
Mean temperature of wettest quarter	19.0734	18.5687	19.0984
Mean temperature of driest quarter	1.8392	2.6540	3.1937
Mean temperature of warmest quarter	19.3620	20.1693	20.1407
Mean temperature of coldest quarter	0.7650	1.0070	1.4554
Annual precipitation	741.9998	584.9998	716.0001
Annual precipitation of wettest month	20.6000	17.1688	21.2420
Precipitation of driest month	8.9749	8.0606	9.2098
Precipitation seasonality (coefficient of variation)	0.2567	0.2096	0.2236
Precipitation of wettest quarter	250.1101	188.0460	232.0261
Precipitation of driest quarter	123.9644	111.8423	129.1972
Precipitation of warmest quarter	246.1459	176.3892	221.8941
Precipitation of coldest quarter	137.7727	124.6560	146.1784

SEO1, Hévíz; SEO2, Homokmégy; SEO3, Vejti.

### 3.3 Analytical composition of *Solidago gigantea* essential oil

The GC–MS analysis of EOs extracted from the inflorescences of *Solidago gigantea* resulted in the detection of 110 compounds (Table 4; Supplementary Figure S1). These constituents were categorized into 7 chemical classes: hydrocarbon monoterpenes (HMT) (12), oxygenated monoterpenes (OMT) (19), hydrocarbon sesquiterpenes (HST) (36), oxygenated sesquiterpenes (OST) (35), hydrocarbon diterpene (HDT) (1), oxygenated diterpene (ODT) (1), and others (6) (Table 4). Essential oils derived from *Solidago gigantea* inflorescences were dominated by oxygenated sesquiterpenes (SEO1: 44.67%, SEO2: 49.72%, SEO3: 58.17%), followed by hydrocarbon monoterpenes (SEO1: 26.02%, SEO2: 21.39%, SEO3: 12.97%) and hydrocarbon sesquiterpenes (SEO1: 19.43%, SEO2: 18.21%, SEO3: 21.87%).

**Table 4:** Semi-quantitative chemical composition of *Solidago gigantea* essential oils using GC–MS.

No	Compound name	Class	LRI_lit_	LRI_exp_	SEO1	SEO2	SEO3
1	**Cyclocolorenone**	OST	1,757	1,760	**10.46**	**13.45**	**29.69**
2	**Gurjunene <alpha->**	HST	1,406	1,409	**2.76**	**3.33**	**6.32**
3	**Pinene <alpha>**	HMT	933	932	**12.41**	**9.90**	**5.09**
4	**Bornyl acetate**	OMT	1,285	1,286	**5.80**	**6.06**	**4.31**
5	**Spathulenol**	OST	1,576	1,579	**2.96**	**3.62**	**4.31**
6	**Germacrene D**	HST	1,480	1,482	**3.98**	**3.27**	**3.77**
7	**Cymene <para->**	HMT	1,025	1,024	**4.38**	**4.51**	**3.15**
8	Gurjunene <gamma->	HST	1,476	1,473	1.30	1.34	2.78
9	Ledol^a^	OST	-	1,604	1.12	1.68	2.77
10	Opposita-4(15)-11-dien-1-ol^a^	OST	-	1,655	2.90	2.74	2.13
11	Mintoxide	OST	1,565	1,567	2.76	2.44	1.86
12	Caryophyllene <(E)->	HST	1,424	1,420	1.34	1.32	1.86
13	Eudesma-4(15)0.7-dien-1beta-ol	OST	1,685	1,688	2.34	3.13	1.73
14	Caryophyllene oxide	OST	1,587	1,584	1.39	1.81	1.59
15	Salvial-4(14)-en-1-one isomer	OST	1,596	1,595	1.72	1.83	1.43
16	Cubenol <1–0.10-di-epi->	OST	1,614	1,614	1.72	1.90	1.39
17	Myrcene	HMT	991	991	2.86	1.12	1.30
18	Muurola-4.10(14)-dien-1-ol	OST	1,632	1,630	3.46	3.46	1.24
19	Pinene <beta->	HMT	978	975	2.32	2.13	1.24
20	Costol <alpha->	OST	1,770	1,767	1.42	1.42	1.01
21	Cadinene <gamma->	HST	1,512	1,515	2.35	2.08	0.94
22	Sabinene	HMT	972	972	1.84	1.51	0.91
23	Germacra-4(15)0.5.10(14)-trien-1-alpha-ol isomer I	OST	1,683	1,678	1.06	0.80	0.84
24	Muurolene <gamma->	HST	1,478	1,477	1.13	1.01	0.79
25	Unknown OS	OST	-	1,593	0.98	1.01	0.79
26	Humulene epoxide II	OST	1,613	1,610	1.01	1.17	0.75
27	Guaia-6.10(14)-dien-4-beta-ol^a^	OST	-	1,619	1.11	0.90	0.73
28	Naphthalene derivative	O	-	1,839	1.41	0.95	0.64
29	Salviadienol	OST	1,545	1,550	0.80	1.04	0.60
30	Camphene	HMT	953	948	0.76	0.96	0.57
31	Cadin-4-en-10-ol	OST	1,659	1,657	0.78	1.03	0.56
32	Calamenene <cis->	HST	1,521	1,523	0.64*	0.68*	0.55*
33	Cadinene <delta->	HST	1,518	1,523			
34	Elemene <beta->	HST	1,390	1,392	0.66	0.36	0.55
35	Valencene	HST	1,492	1,495	0.43	0.40	0.51
36	Limonene	HMT	1,030	1,028	1.04	0.83	0.49
37	Opposita-4(15)-7(11)-dien-1-ol isomer^a^	OST	-	1,648	0.77	0.77	0.48
38	Germacra-4(15)0.5.10(14)-trien-1-alpha-ol isomer II	OST	1,683	1,682	0.76	0.70	0.48
39	Eudesma-4(15)0.7-dien-1beta-ol isomer^a^	OST	-	1,673	0.55	0.42	0.47
40	Palustrol	OST	1,568	1,569	0.19	0.32	0.46
41	Selinene <beta->	HST	1,492	1,487	0.52	0.56	0.42
42	Copaene <beta->	HST	1,433	1,430	0.44	0.38	0.42
43	Humulene <alpha->	HST	1,454	1,454	0.50	0.35	0.37
44	Indane derivative	O	-	2,103	0.22	0.35	0.36
45	Unknown OST C14H22O	OST	-	1,689	0.69	0.63	0.35
46	Cyclocolorenone <epi->	OST	1,771	1,776	0.25	0.38	0.34
47	Unknown OS	OST	-	1,746	0.68	0.37	0.34
48	Cubenol <10-epi->^a^	OST	-	1,624	0.47	0.48	0.33
49	Verbenol <trans->	OMT	1,145	1,145	0.48	0.66	0.31
50	Muurolene <15-oxy-alpha->	OST	1,765	1,770	0.38	0.48	0.30
51	Salvial-4(14)-en-1-one isomer	OST	1,596	1,592	0.38	0.39	0.28
52	beta-Spathulene	HST	1,457	1,452	0.05	0.11	0.27
53	Phytone	O	1,841	1,845	0.17	0.31	0.25
54	Opposita-4(15)-7(11)-dien-1-ol	OST	1,633	1,633	0.25	0.27	0.24
55	Cadinene <alpha->	HST	1,538	1,539	0.61	0.52	0.23
56	Muurolene <alpha->	HST	1,497	1,501	0.30	0.25	0.22
57	Guaiene <alpha->	HST	1,438	1,442	0.08	0.11	0.19
58	Unknown OST C16H28O	OST	-	1,801	0.24	0.21	0.17
59	Isogermacrene D	HST	1,447	1,445	0.18	0.14	0.15
60	Cubebene <6-epi-alpha->	HST	1,418	1,414	0.07	0.07	0.15
61	Muurolol <alpha-. epi->	OST	1,645	1,644	0.44	0.33	0.13
62	Bourbonene <beta	HST	1,382	1,385	0.16	0.12	0.13
63	7-epi-Eremophila-1(10)0.8.11-triene	HST	1,508	1,505	0.06	0.07	0.13
64	Bergamotene <alpha-. trans->	HST	1,432	1,436	0.43	0.36	0.12
65	Terpinen-4-ol	OMT	1,184	1,179	0.14	0.18	0.12
66	Myrtenal	OMT	1,197	1,197	0.15	0.26	0.11
67	Thujene <alpha->	HMT	926	927	0.16	0.20	0.11
68	Unkown OS	OST	-	1,906	0.26	0.12	0.11
69	Amorphene <epsylon->	HST	1,502	1,498	0.12	0.09	0.11
70	Campholenal <alpha->	OMT	1,125	1,127	0.15	0.27	0.10
71	Cubebene <beta->	HST	1,392	1,390	0.21	0.16	0.10
72	Calamenen-10-one <10-nor->	OST	1,701	1,705	0.04	0.15	0.10
73	Calacorene <alpha->	HST	1,544	1,544	0.10	0.12	0.10
74	Alloaromadendrene	HST	1,458	1,461	0.01	0.04	0.10
75	Pinocarveol <trans->	OMT	1,141	1,138	0.12	0.24	0.09
76	Muurola-4(14)0.5-diene <cis->	HST	1,466	1,463	0.22	0.23	0.09
77	Verbenone	OMT	1,208	1,210	0.15	0.21	0.09
78	Copaene <alpha->	HST	1,375	1,376	0.13	0.12	0.09
79	Nonene <n->	O	890	890	0.12	0.15	0.08
80	Cadinol <τ->	OST	1,641	1,642	0.16	0.13	0.08
81	Bourbon-11-ene	HST	1,424	1,423	0.05	0.06	0.08
82	Cubebene <alpha->	HST	1,355	1,350	0.10	0.11	0.07
83	Furan <2-butyl->	O	890	885	0.17	0.10	0.06
84	Elemene <delta->	HST	1,335	1,336	0.11	0.10	0.06
85	Ylangene <alpha->	HST	1,371	1,371	0.10	0.10	0.06
86	Calacorene <beta->	HST	1,564	1,564	0.07	0.08	0.06
87	Linoleic acid	O	2,144	2,144	0.04	0.05	0.06
88	Torilenol	OST	1,599	1,599	0.15	0.14	0.05
89	Elemene <gamma->	HST	1,432	1,434	0.17	0.12	0.05
90	Thuja-2.4(10)-diene	HMT	953	952	0.10	0.12	0.05
91	Kaurene	HDT	2,045	2,040	0.10	0.10	0.05
92	Cymen-8-ol <para->	OMT	1,189	1,187	0.05	0.10	0.05
93	Manoyl oxide <13-epi->	ODT	2,017	2,015	0.04	0.05	0.05
94	Verbenol <cis->	OMT	1,141	1,141	0.08	0.09	0.04
95	Viridiflorol	OST	1,594	1,594	0.02	0.00	0.04
96	Perillene	OMT	1,098	1,102	0.09	0.07	0.03
97	Sabina ketone <dehydro->	OMT	1,122	1,121	0.05	0.06	0.03
98	Linalool	OMT	1,101	1,101	0.03	0.06	0.03
99	Menthatriene <1.3.8-para->	HMT	1,106	1,106	0.09	0.05	0.03
100	Borneol	OMT	1,165	1,166	0.04	0.05	0.03
101	Pinene <alpha-> epoxide (Isomer 1)	OMT	1,096	1,091	0.05	0.07	0.02
102	Terpineol <alpha->	OMT	1,195	1,192	0.05	0.07	0.02
103	Tricyclene	HMT	923	921	0.04	0.04	0.02
104	Thujone <beta->	OMT	1,118	1,117	0.04	0.03	0.02
105	Isoledene	HST	1,372	1,377	0.01	0.01	0.02
106	Cyclosativene	HST	1,367	1,367	0.04	0.04	0.01
107	Pinene <alpha-> epoxide (Isomer 2)	OMT	1,101	1,098	0.03	0.03	0.01
108	Camphenone <6->	OMT	1,099	1,096	0.02	0.03	0.01
109	Carveol <trans->	OMT	1,223	1,221	0.02	0.03	0.01
110	Phellandrene <alpha->	HMT	1,007	1,005	0.02	0.02	0.01
	Total				99.93	99.95	99.99
	Hydrocarbon monoterpenes (HMT)				26.02	21.39	12.97
	Oxygenated monoterpenes (OMT)				7.54	8.57	5.43
	Hydrocarbon sesquiterpenes (HST)				19.43	18.21	21.87
	Oxygenated sesquiterpenes (OST)				44.67	49.72	58.17
	Hydrocarbon diterpenes (HDT)				0.10	0.10	0.05
	Oxygenated diterpenes (ODT)				0.04	0.05	0.05
	Others (O)				2.13	1.91	1.45

SEO1, Hévíz; SEO2, Homokmégy; SEO3, Vejti.

HMT, hydrocarbon monoterpenes; OMT, oxygenated monoterpenes; HST, hydrocarbon sesquiterpenes; OST, oxygenated sesquiterpenes; HDT, hydrocarbon diterpenes; ODT, oxygenated diterpenes; O, others.

^*^Co-eluted compounds, ^a^Tentative identification based on Kalemba et al. (2001).

Bold text indicates the major constituents (main components) of the essential oil samples.

Although the same components were identified in EOs from all three samples, their relative quantities differed considerably. Specifically, the cyclocolorenone content in SEO3 was nearly threefold higher, and the *α*-gurjunene content approximately twofold higher, than those detected in SEO1. In contrast, α-pinene was most abundant in the SEO1 sample. Other prominent constituents included spathulenol, bornyl acetate, germacrene D, and p-cymene.

### 3.4 Microbiological results

#### 3.4.1 Minimum inhibitory concentration

Based on the determination of MIC, SEOs exhibited antibacterial activity against all tested pathogens. *E. coli* was the most sensitive to the treatment by SEOs, as indicated by the lowest MIC values, while *P. aeruginosa* showed the highest resistance. The SEO3 demonstrated the strongest antibacterial activity across all bacterial strains. Nevertheless, the antibiotics used as positive controls were more effective than the SEOs against each tested strain (Table 5).

**Table 5:** The minimum inhibitory concentrations (mg/mL) of *Solidago gigantea* essential oils and antibiotics.

Treatment	*E. coli*	MRSA	*P. aeruginosa*
SEO1	0.312	1.250	2.500
SEO2	0.625	1.250	2.500
SEO3	0.312	0.625	1.250
Antibiotics	0.004	0.013	0.006

SEO1, Hévíz; SEO2, Homokmégy; SEO3, Vejti. Antibiotics: *E. coli*: ceftriaxone, MRSA and *P. aeruginosa*: gentamicin.

#### 3.4.2 Biofilm inhibition

**Figure 2:**
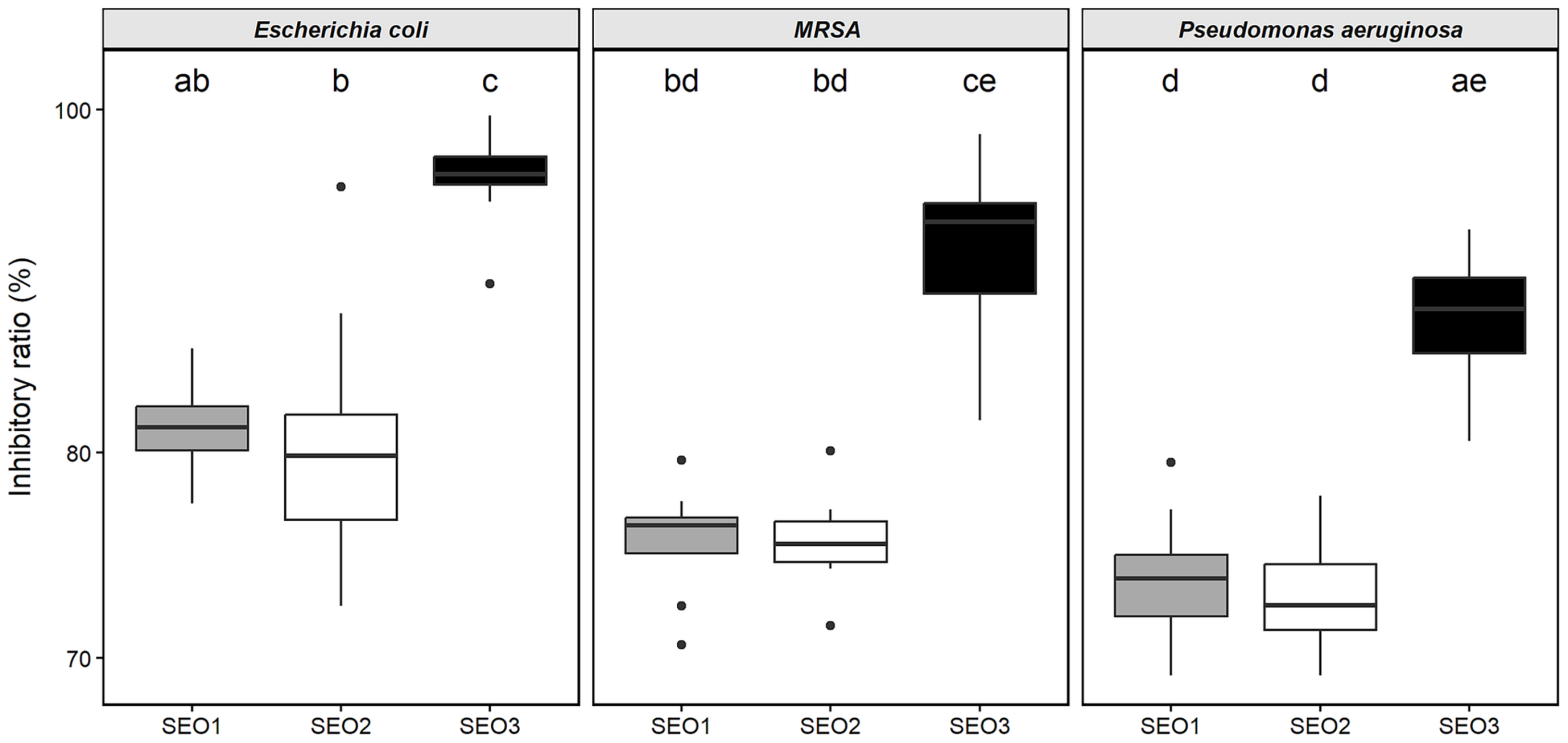
Inhibitory rate of essential oils derived from *Solidago gigantea* inflorescences. SEO1, Hévíz; SEO2, Homokmégy; SEO3, Vejti.

The CV assay demonstrated that SEOs were effective in inhibiting biofilm formation in all tested bacterial strains. *E. coli* was the most susceptible, showing biofilm inhibition rates of 81.32, 80.77, and 95.70% for SEO1, SEO2, and SEO3, respectively. Similar levels of inhibition were observed for MRSA, with values of 75.61, 75.57, and 91.16%, respectively. *P. aeruginosa* was the most resistant strain, exhibiting biofilm reduction rates of 73.77, 72.95, and 87.35% for the respective samples. Statistical analysis confirmed that SEO3 exhibited the highest antibiofilm activity, with no significant difference detected between SEO1 and SEO2 (Figure 2).

#### 3.4.3 Membrane degradation

Membrane degradation assays revealed that MIC/4 and MIC/2 concentrations did not result in measurable membrane degradation after 60 min. In contrast, treatment at the MIC concentration demonstrated considerable and statistically significant efficacy, with all three SEOs causing membrane damage across all tested bacterial strains (*E. coli*: *p* < 0.001, MRSA: *p* < 0.001, *P. aeruginosa*: *p* < 0.001). The SEO3 exhibited the highest activity against *E. coli*, with a membrane degradation rate of 84.07% at MIC. A concentration-dependent increase in membrane disruption was observed, with *E. coli* consistently emerging as the most sensitive strain and SEO3 as the most effective sample. At MIC×2 concentration, SEO3 induced 91.3% membrane damage in *E. coli*, and notably, membrane damage exceeding 80% was also recorded for *P. aeruginosa*, the most resistant strain (Table 6).

**Table 6:** Membrane degradation rate and *Solidago gigantea* essential oils concentration dependence (%), time: 60 min, *n* = 8.

Concentration	SEO samples	*E. coli*	MRSA	*P. aeruginosa*
MIC/2	SEO1	0.00 ^a^	0.00 ^a^	0.00 ^a^
	SEO2	0.00 ^a^	0.00 ^a^	0.00 ^a^
	SEO3	0.00 ^a^	0.00 ^a^	0.00 ^a^
MIC	SEO1	75.01 ± 2.8^b^	70.11 ± 4.3^b^	64.97 ± 3.6^b^
	SEO2	67.61 ± 3.3^c^	62.47 ± 3.2^c^	55.25 ± 3.6^c^
	SEO3	84.07 ± 3.7^d^	81.80 ± 1.2^d^	75.47 ± 3.7^d^
MIC×2	SEO1	82.02 ± 4.5^d^	78.37 ± 3.8^d^	73.85 ± 3.0^d^
	SEO2	74.16 ± 2.5^b^	68.43 ± 3.2^b^	64.41 ± 4.1^b^
	SEO3	91.37 ± 15^e^	87.53 ± 2.2^e^	84.30 ± 2.9^e^
MIC×4	SEO1	100.00^f^	100.00^f^	100.00^f^
	SEO2	100.00^f^	100.00^f^	100.00^f^
	SEO3	100.00^f^	100.00^f^	100.00^f^

SEO1, Hévíz; SEO2, Homokmégy; SEO3, Vejti.

Lowercase letters indicate significant differences.

Time-dependent experiments conducted at MIC×2 concentration showed that membrane damage was detectable as early as 10 min post-treatment in all bacterial strains. In most cases, damage ranged between 20–30% at this early time point, whereas *E. coli* and MRSA exhibited more pronounced effects, with membrane damage exceeding 40% following treatment with SEO3 (Table 7).

**Table 7:** Interrelationship between membrane degradation and time dependence (%), concentration: MIC×2, *n* = 8.

Time (min)	SEO samples	*E. coli*	MRSA	*P. aeruginosa*
10	SEO1	34.32 ± 2.6^a^	30.61 ± 1.7^a^	29.22 ± 2.4^a^
	SEO2	29.08 ± 3.2^a^	26.20 ± 3.5^a^	24.47 ± 3.8^a^
	SEO3	45.92 ± 0.8^b^	41.08 ± 3.6^bc^	35.25 ± 2.2^b^
20	SEO1	64.77 ± 6.9^c^	45.31 ± 3.0^b^	40.27 ± 2.4^b^
	SEO2	44.80 ± 3.1^b^	39.71 ± 2.8^c^	35.08 ± 2.9^b^
	SEO3	74.57 ± 4.4^d^	55.33 ± 2.0^d^	55.05 ± 2.1^c^
60	SEO1	82.02 ± 4.5^e^	78.37 ± 3.8^e^	73.85 ± 3.0^d^
	SEO2	74.16 ± 2.5^d^	68.43 ± 3.2^f^	65.41 ± 4.1^e^
	SEO3	91.37 ± 1.5^f^	87.53 ± 2.2^g^	84.30 ± 2.9^f^
90	SEO1	97.21 ± 2.4^g^	95.21 ± 2.4^h^	92.86 ± 3.7^g^
	SEO2	97.45 ± 1.9^g^	93.42 ± 6.1^h^	87.15 ± 5.6^f^
	SEO3	99.78 ± 0.4^g^	98.00 ± 1.1^h^	95.72 ± 1.6^g^

SEO1, Hévíz; SEO2, Homokmégy; SEO3, Vejti.

Lowercase letters indicate significant differences.

#### 3.4.4 Synergistic effect

To evaluate the potential application of SEO in preventive and/or adjunctive therapy, a checkerboard titration assay was performed. The combination tests were performed with SEO3, which demonstrated the highest efficacy in previous microbiological assays. Based on the fractional inhibitory concentration index (FICI) values calculated from the combination MIC values, a synergistic interaction was observed between gentamicin and SEO3 against *P. aeruginosa*. A similar synergistic effect was detected between ceftriaxone and SEO3 in *E. coli*. Furthermore, an additive effect was observed between vancomycin and SEO3 against MRSA (Table 8).

**Table 8:** The quality of the interaction between *Solidago gigantea* essential oil and antibiotics.

Bacterial strain	Samples	Combination MIC	FICI	Quality of interaction
*E. coli*	SEO3	0.0780	0.37	**Synergistic**
	cefrtiaxone	0.0005		
MRSA	SEO3	0.1560	0.75	Additive
	vancomycin	0.0065		
*P. aeruginosa*	SEO3	0.3125	0.50	**Synergistic**
	gentamicin	0.0015		

SEO3, Vejti.

Bold text highlights the synergistic interaction (synergy) observed in the checkerboard assay (based on FICI interpretation).

## 4 Discussion

Investigating the antibacterial and biofilm-inhibitory properties of natural compounds is crucial for developing novel therapeutic strategies to address the escalating problem of antibiotic resistance. In this context, the antimicrobial potential of EOs and their constituents has been extensively studied. Due to their complex chemical composition, EOs represent a promising approach to mitigate antibiotic resistance (Kon and Rai, 2016).

The chemical composition of *Solidago gigantea* inflorescence EOs analyzed in this study was primarily characterized by oxygenated sesquiterpenes (44.67–58.17%) and hydrocarbon monoterpenes (12.97–26.02%), followed by hydrocarbon sesquiterpenes (19.43–21.87%) and oxygenated monoterpenes (5.43–8.57%). These results are consistent with previous phytochemical analyses of SEOs, which similarly emphasized the predominance of these compound classes (Benelli et al., 2019; Nkuimi Wandjou et al., 2020; Radušienė et al., 2022). Using multivariate data analysis, Radušienė et al. (2022) further elucidated the interspecies relationships within the genus *Solidago*, revealing considerable similarity in EO profiles predominantly composed of monoterpenes and oxygenated compounds. Nonetheless, species-specific variations were noted in the occurrence and distribution of distinctive terpenes across different accessions and plant parts (Radušienė et al., 2022). In agreement with our results, *α*-pinene, cyclocolorenone, bornyl acetate, germacrene D, and spathulenol have previously been reported as major constituents of SEOs (Kalemba et al., 2001; Kołodziej et al., 2011; Benelli et al., 2019). For example, Kalemba et al. (2001) investigated a Polish population of *Solidago gigantea* and identified germacrene D (23.5%) and cyclocolorenone (32.4%) as the predominant components of EOs obtained from aerial parts. In contrast, Radušienė et al. (2022), in their comparative assessment of volatile compound distribution across four *Solidago* species, did not detect cyclocolorenone in the EO of *Solidago gigantea*.

All 110 identified compounds were consistently present in the SEOs, however, the relative quantities of certain components varied among samples. In SEO3, originating from the warmest collection site, concentration of cyclocolorenone was approximately three times higher, while the α-pinene content was reduced by nearly 50% compared to the other two populations. Such variations may be attributed to a range of biotic and abiotic factors influencing secondary metabolite biosynthesis. Increasing evidence suggests that both the yield and chemical profile of EOs are shaped by environmental factors such as temperature, light exposure, precipitation, and altitude (Barra, 2009). Figueiredo et al. (2008) reported that monoterpene emission in plants increases with rising temperatures within the range of 20–46 °C. Consequently, light and solar radiation represent critical factors in the biosynthesis of EOs. Moreover, under drought conditions, plants have been shown to enhance monoterpene production as a protective response to mitigate oxidative stress induced by reactive oxygen species (Nowak et al., 2010).

In our study, SEOs demonstrated notable antibacterial activity against all tested pathogenic strains. Among the Gram-negative strains tested, *E. coli* exhibited the lowest resistance to the analyzed samples of SEOs, while *P. aeruginosa* showed the highest one. The resistance of the Gram-positive MRSA was intermediate relative to these two Gram-negative species. Gram-positive bacteria are more affected by EO components with hydrophobic properties (Plésiat and Nikaido, 1992; Angane et al., 2022). This phenomenon can be explained by the fact that Gram-positive bacteria have a thick peptidoglycan layer linked to apolar molecules, through which lipophilic components can easily pass, whereas Gram-negative bacteria are prevented from doing so by the cell envelope, outer membrane-attached proteins and lipopolysaccharides (Nikaido, 1994). Despite being a Gram-negative bacterium, we found that *E. coli* is the least resistant to SEOs because *E. coli* possesses a relatively permeable outer membrane compared to other Gram-negative bacteria, such as *P. aeruginosa*. While the lipopolysaccharide (LPS) layer normally provides a strong barrier against hydrophobic molecules, studies have shown that *E. coli* outer membrane porins allow an enhanced passive diffusion of small hydrophobic and amphipathic molecules (Nikaido and Vaara, 1985; Delcour, 2009). This antimicrobial effect is likely primarily associated with the presence of key bioactive constituents, including cyclocolorenone, spathulenol, and germacrene D. Cyclocolorenone, classified as an aromadendrene-type sesquiterpene, has been well-documented for its potent phytotoxic, antibacterial, and antifungal properties against a range of microorganisms, including *Bacillus subtilis*, *B. cereus*, *Microbacterium thermosphactum*, *Escherichia coli*, *Enterobacter cloacae*, *Citrobacter freundii*, *Curvularia lunata*, and *Cochliodes spinusum* (Jacyno et al., 1991). Cyclocolorenone has been previously isolated from various plant species such as *Solidago canadensis* (Schmidt et al., 1999), *Magnolia grandiflora* (Jacyno et al., 1991), and *Critonia aromatisans* (la Torre Fabiola et al., 2016). On the other hand, the presence cyclocolorenone in *Solidago virgaurea* EO has not been reported (Bertoli et al., 1999). Spathulenol, an oxygenated sesquiterpene, was identified in concentrations ranging from 2.96 to 4.31% across the analyzed samples. This compound has great antimicrobial and immunomodulatory potential (Ziaei et al., 2011; Cazella et al., 2019). Germacrene D is a monocyclic sesquiterpene commonly found in various EOs. It can be a precursor to many other sesquiterpenes and possesses a wide range of biological activity, including substantial antimicrobial activity (Şahin et al., 2004; Liu et al., 2022).

Many plant-derived natural products have been shown to exert anti-biofilm effects *in vitro* (Lu et al., 2019; Tsukatani et al., 2020). However, limited data are available regarding the biofilm inhibitory activity of *Solidago* species, with existing studies primarily focusing on *Solidago virgaurea* (Chevalier et al., 2019; Abdulkareem et al., 2023). To the best of our knowledge, the present study is the first to demonstrate anti-biofilm properties of EO from *Solidago gigantea* inflorescences. *Solidago gigantea* EOs were effective in inhibiting biofilm formation in all tested bacterial strains. *P. aeruginosa* was the most resistant pathogen. This may be due to the fact that this bacterium has three exopolysaccharides: Psl, Pel, and alginate. Psl is a neutral pentasaccharide that typically contains D-glucose, D-mannose, and L-rhamnose moieties. Psl acts as a signaling molecule and promotes the production of c-di-GMP (bis-(3′-5′)-cyclic dimeric guanosine monophosphate), the levels of which, when elevated, result in thicker and more robust biofilms (Irie et al., 2012). The other pathogens included in the study (*E. coli*, MRSA) do not have such resistant biofilms, so higher inhibition rate values were detected for these pathogens.

Based on their chemical composition, the antimicrobial and antibiofilm activities of SEOs are likely attributable to the combined, and potentially synergistic, effects of their major sesquiterpene and monoterpene constituents. Both oxygenated and hydrocarbon sesquiterpenes, including cyclocolorenone, spathulenol, and germacrene D, are highly lipophilic compounds that readily interact with bacterial cell envelopes. Such interactions may increase membrane permeability, disrupt membrane integrity, and promote leakage of intracellular components, ultimately leading to impaired energy metabolism and enzyme dysfunction (Bakkali et al., 2008; Nazzaro et al., 2013; Mishra et al., 2020). In addition to their antimicrobial activity, these compounds may also interfere with biofilm development through multiple mechanisms, including inhibition of initial cell adhesion, alteration of cell surface hydrophobicity, and disruption of extracellular polymeric substance (EPS) production (Lu et al., 2019; Das, 2022). Sesquiterpenes have further been reported to modulate quorum sensing-regulated pathways and intracellular signaling systems, including cyclic di-GMP, which are central to biofilm maturation and stability, particularly in Gram-negative pathogens such as *P. aeruginosa* (Kostakioti et al., 2013; Melander et al., 2020). Accordingly, the pronounced antibiofilm activity observed in the present study is likely driven by a multifactorial mode of action that combines direct antimicrobial effects with the modulation of key biofilm-associated virulence mechanisms.

In addition to their antimicrobial properties, SEOs may act synergistically with conventional antibiotics, thereby enhancing their efficacy against antibiotic-resistant bacteria (Langeveld et al., 2014; Magi et al., 2015). In our study the activity of SEO was evaluated in combination with ceftriaxone, gentamicin, and vancomycin against *E. coli*, *P. aeruginosa*, and MRSA, respectively. The combination assays demonstrated that SEO not only enhances antibiotic efficacy through biofilm inhibition but also exerts additive or synergistic effects. Notably, synergistic interactions were observed for ceftriaxone with SEO against *E. coli* and for gentamicin with SEO against *P. aeruginosa*. Our findings are partially in agreement with previous reports, where the combination of gentamicin and thyme (*Thymus maroccanus*) EO exhibited a synergistic effect against *P. aeruginosa* and *E. coli* (Fadli et al., 2012), whereas the combination of ceftriaxone and oregano (*Origanum vulgare*) EO resulted in an additive interaction (Si et al., 2008). The markedly lower MIC values of standard antibiotics compared to SEOs were expected, as antibiotics are single, highly optimized antimicrobial agents, whereas EOs are complex natural mixtures. Therefore, the comparison serves as a benchmark reference rather than a claim of equivalent antibacterial potency. In this context, the proposed relevance of SEOs lies primarily in their antibiofilm activity and their potential use as adjunctive agents rather than as standalone antimicrobial therapies.

Although the present study highlights the pronounced antibacterial and antibiofilm activities of SEOs, their safety profile was not assessed. Future work should investigate cytotoxicity, hemolytic activity, and potential irritation effects on host tissues to establish the therapeutic window and ensure safe application, particularly for topical or adjunctive antimicrobial formulations.

## 5 Conclusion

Our results demonstrated that the EO obtained from *Solidago gigantea* inflorescences possesses antibacterial and biofilm-inhibitory activities. The geographical origin of the plant was found to influence not only the quantitative composition of the bioactive constituents, but also its antibacterial and anti-biofilm efficacy.

## Data Availability

The raw data supporting the conclusions of this article will be made available by the authors, without undue reservation.
