# Supplementary material for: Geographical variation in the chemical profile and antimicrobial activity of Solidago gigantea essential oils
Source: Front Microbiol. 2026 Feb 9;17:1740133. doi: 10.3389/fmicb.2026.1740133 (PMC12926419; doi:10.3389/fmicb.2026.1740133)
Supplement: Supplementary file 1 [file Table_1.docx]

**Supplementary materials**

S1. Minimum inhibitory concentration (MIC) values of *Solidago gigantea* essential oils and corresponding MIC-derived concentrations used in antibiofilm and membrane degradation assays

|  |  | *E. coli* | MRSA | *P. aeruginosa* |
| --- | --- | --- | --- | --- |
| MIC/4 | SEO1 | 0.078 | 0.313 | 0.625 |
|  | SEO2 | 0.156 | 0.313 | 0.625 |
|  | SEO3 | 0.078 | 0.156 | 0.313 |
| MIC/2 | SEO1 | 0.156 | 0.625 | 1.250 |
|  | SEO2 | 0.313 | 0.625 | 1.250 |
|  | SEO3 | 0.156 | 0.313 | 0.625 |
| MIC | SEO1 | 0.312 | 1.250 | 2.500 |
|  | SEO2 | 0.625 | 1.250 | 2.500 |
|  | SEO3 | 0.312 | 0.625 | 1.250 |
| MIC×2 | SEO1 | 0.624 | 2.500 | 5.000 |
|  | SEO2 | 1.250 | 2.500 | 5.000 |
|  | SEO3 | 0.624 | 1.250 | 2.500 |
| MIC×4 | SEO1 | 1.248 | 5.000 | 10.000 |
|  | SEO2 | 2.500 | 5.000 | 10.000 |
|  | SEO3 | 1.248 | 2.500 | 5.000 |

SEO1: Hévíz, SEO2: Homokmégy, SEO3: Vejti

S2. Rapresentative GC-MS total ion current (TIC) chromatogram of *Solidago gigantea* (SEO1)

**
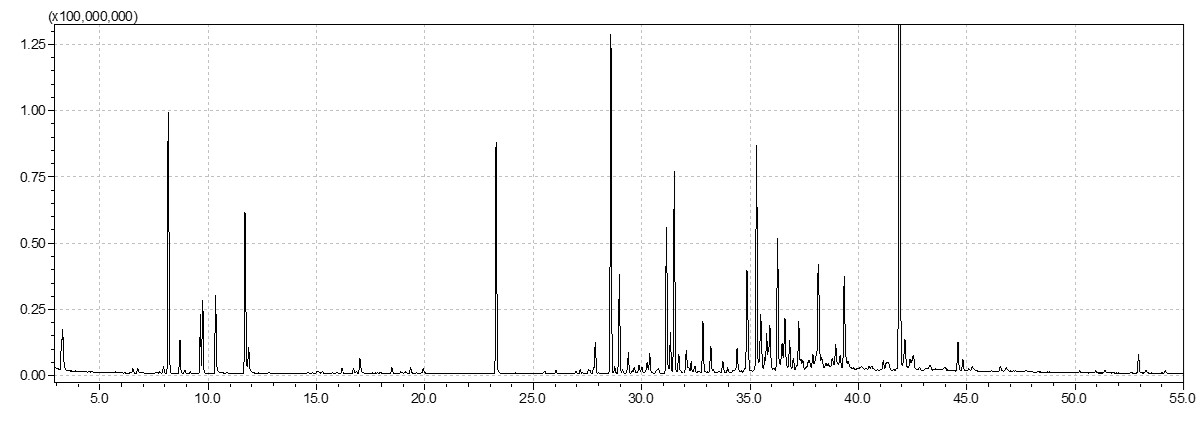
**

SEO1: Hévíz
